# *Enterococcus casseliflavus* regulates amino acid metabolism in edible insect *Clanis bilineata tsingtauica*: a functional metagenomics study

**DOI:** 10.3389/fmicb.2024.1343265

**Published:** 2024-03-25

**Authors:** Lei Qian, Yanhui Wang, Pan Deng, Jia Zhang, Yi Qin, Zongnan Li, Huaijian Liao, Fajun Chen

**Affiliations:** ^1^Institute of Leisure Agriculture, Jiangsu Academy of Agricultural Sciences, Nanjing, China; ^2^College of Plant Protection, Nanjing Agricultural University, Nanjing, China; ^3^College of Plant Protection, Yangzhou University, Yangzhou, China; ^4^College of Biotechnology, Jiangsu University of Science and Technology, Zhenjiang, China

**Keywords:** edible insect, *Clanis bilineata tsingtauica*, metagenomics, dominant gut bacterium, nutrient metabolism

## Abstract

**Introduction:**

The soybean hawkmoth, *Clanis bilineata tsingtauica*, is an edible insect that possesses high nutritional, medicinal and economic value. It has developed into a characteristic agricultural industry in China.

**Methods:**

The dominant gut bacterium in diapause larvae of soybean hawkmoths was identified by metagenomics, and the effect of diapause time on gut microbiome composition, diversity and function was investigated.

**Results:**

*Enterococcus* and *Enterobacter* were measured to be the dominant genera, with *Enterococcus casseliflavus* and *Enterococcus pernyi* being the dominant species. Compared to the controls, the relative abundance of *E. casseliflavus* and *E. pernyi* on day 14 was lower by 54.51 and 42.45%, respectively. However, the species richness (including the index of Chao and ACE) of gut microbiota increased on day 28 compared to controls. The gene function was mainly focused on carbohydrate and amino acid metabolism. Metabolic pathways annotated for amino acids on day 14 increased by 9.83% compared to controls. It is speculated that diapause soybean hawkmoths may up-regulate amino acid metabolism by reducing *E. casseliflavus* abundance to maintain their nutritional balance. Additionally, tetracycline, chloromycetin and ampicillin were screened as the top three antibiotics against *E. casseliflavus*.

**Discussion:**

This study not only extends our knowledge of gut microbiome in soybean hawkmoths at the species level, but also provides an initial investigation of gene functionality in interaction with insect hosts.

## 1 Introduction

In China, there is a unique edible insect species *Clanis bilineata tsingtauica*, soybean hawkmoth, which belongs to the Sphingidae family of Lepidoptera (Zhu and Wang, 1997). The larvae of soybean hawkmoth have high nutritional, medicinal, and economic value with abundant protein, vitamins, amino acids (especially essential amino acids (EAAs)), unsaturated fatty acids, microelements, chitin, etc. (Tian and Zhang, 2012). These nutrients can help people promote brain development, prevent the cell deterioration and maintain the endocrine balance (Qian et al., 2022). Currently, in the rural areas of China, especially in the north of Jiangsu Province, the artificial breeding of soybean hawkmoth has become a characteristic agricultural industry with an output of about 30,000 tons and 4.5 billion yuan every year (Guo et al., 2021). Under natural conditions, one generation of the soybean hawkmoth occurs each year in China. The old fifth-instar larvae burrow into the soil and keep still in early September, entering the diapause stage (Guo et al., 2021; Qian et al., 2023a).

Many insect species adaptively choose diapause, similar to hibernation or dormancy in mammals, to increase survival under adverse environmental conditions [e.g., low temperature, lack of food and water (Dittmer and Brucker, 2021)]. It is critical to provide adequate nutrition for diapause insects, and many species build up nutrient reserves in advance. Despite entering a stage of developmental arrest, diapause insects are able to maintain a stable body weight and nutritional status (Santana et al., 2017). However, the nutrient composition of some insects also varies with the intensity of diapause. Examples include lipids in diapause *Arimania comaroffi*, and glycerol, trehalose and glycogen in diapause *Aphidius gifuensis* (Bemani et al., 2012). During diapause, insects can self-regulate the composition and levels of nutrients, such as protein, fatty acids, EAAs and carbohydrates (Hahn and Denlinger, 2011). In addition to nutrient storage, insects also reduce their metabolism in order to meet energy requirements for diapause and to provide energy sources for post-diapause growth and development (Poelchau et al., 2013; Dittmer and Brucker, 2021). Despite the growing interest of entomologists in the regulatory mechanisms of insect diapause, the interaction between gut microbes and diapause insects, especially in aspects of nutrient and metabolic regulation, is still poorly understood (Engel and Moran, 2013; Douglas, 2015).

In numerous insect species, gut microbes are involved in nutrient metabolism, digestion, gut homeostasis and endocrine signaling to promote their development and growth, as noted by Zheng et al. (2017) and van Opstal and Bordenstein (2019). For instance, gut microbiota can provide the host insects with a steady supply of essential nutrients, including EAAs, carbohydrates and lipids, to sustain their metabolic processes (Fraune and Bosch, 2010). The gut microbiota assists in fixing atmospheric nitrogen and promoting amino acid metabolism, leading to a partial shift in the host’s amino acid composition pattern (Storelli et al., 2011). In addition, gut bacteria contribute significantly to lipid and protein digestion in *Tenebrio molitor* and *Anticarsia gemmatalis* (Genta et al., 2006; Visotto et al., 2009). They also exhibit enzymatic action on starch, lipids, pectin and esters in *Helicoverpa armigera*, *Bombyx mori*, *Plutella xylostella* and *Antheraea assamensis* (Anand et al., 2010; Gandotra et al., 2018). In *Riptortus pedestris*, the presence of *Burkholderia* in the gut results in higher levels of juvenile hormone (JH), leading to increased fecundity and storage proteins (Lee et al., 2019). Moreover, it has been demonstrated that *Klebsiella oxytoca* can boost the developmental stage of *Drosophila suzukii* by activating the glycolysis pathway (Gao et al., 2023). The gut microbiota in *Drosophila melanogaster* modulates the insulin signaling pathway, a key regulator of insect growth and nutrient homeostasis, to regulate nutrient allocation in the host (Shin et al., 2011).

In our previous study, 16S rRNA sequencing was conducted to analyze the gut microbes of diapause soybean hawkmoths. The results showed that *Proteobacteria* and *Firmicutes* were the most prominent taxa at the phylum level in the larval gut microbiota, with *Enterococcus* and *Stenotrophomonas* being the dominant genera. In addition, non-target metabolomics analysis on the intestinal contents showed that the most enriched pathways was mainly focused on amino acid metabolism (Qian et al., 2023b). Our findings suggest that the dominant gut bacteria may be involved in the nutritional physiology of insect hosts. However, the specific dominant strains of diapause soybean hawkmoths are still unidentified, not to mention their regulatory mechanisms for nutrient biosynthesis and metabolism in the host. This study identified the predominant strains within the larval gut via the metagenomic sequencing, and investigated the impact of diapause time on the diversity, species composition and gene functions of the gut microbiome. This study reports the first discovery of the dominant gut bacteria of soybean hawkmoths, as well as their influence on the nutrition of the hosts. Additionally, antimicrobial susceptibility tests were conducted for the most effective antibiotic to target the dominant bacterium in diapause larvae. Understanding how dominant gut bacteria regulate nutrient synthesis and metabolism in the soybean hawkmoth will help to explore and utilize the nutritional substances of edible insects, promote the development of the soybean hawkmoth industry in China’s rural areas, and further advance the implementation of the rural revitalization strategy.

## 2 Materials and methods

### 2.1 Insects and experimental design

In this study, the population of soybean hawkmoth *C. bilineata tsingtauica* was sourced from Yuntai Farm of Jiangsu Agricultural Reclamation Group, situated in Lian Yun-gang City in Jiangsu Province, China. The soybean hawkmoths were reared on soybean plant leaves at 27°C/16 h light, and 25°C/8 h dark with 70% relative humidity (RH). Once the mature fifth instar larvae start to burrow and cease movement in the soil, they are deemed to have entered the diapause phase. The day when mature larvae entered the soil was recorded as day 0 (considered as the control). Based on our previous study utilizing 16S rRNA sequencing analysis (Qian et al., 2023b), the diapause larvae of soybean hawkmoth at day 0, 14 and 28 were selected for the following metagenomic analysis. Only diapause larvae showing signs of vitality or movement upon stimulation were considered viable. Live larvae were collected for subsequent DNA extraction. The efficient antibiotics were evaluated against the dominant strains identified through metagenomic analysis.

### 2.2 Midgut contents collection

Diapause larvae were collected at various time points (day 0 (D0), day 14 (D14) and day 28 (D28)) and dissected afterward. After cleansing the larvae in a 75% ethanol solution for 2 min, they were rinsed thrice with sterile water to eliminate surface contaminants, and then individually dissected using aseptic forceps and scissors. The midguts were aseptically sampled to obtain intestinal contents, rapidly frozen in liquid nitrogen, and stored at −80°C following the protocol described by Chen et al. (2023). Metagenomic analysis was performed on the intestinal contents collected from diapause larvae. One sample contained the intestinal contents from 20 larvae, with three experimental repeats.

### 2.3 Metagenome DNA extraction and shotgun sequencing

The total microbial genomic DNA samples were extracted using the OMEGA Mag-Bind Soil DNA Kit (M5635-02; Omega Bio-Tek, Norcross, GA, United States) and were kept stored at −20°C for subsequent evaluation. The quantity and quality of extracted DNA were analyzed with the Qubit™ 4 Fluorometer, with WiFi: Q33238 (Qubit™ Assay Tubes: Q32856; Qubit™ 1X dsDNA HS Assay Kit: Q33231; Invitrogen, USA) and agarose gel electrophoresis. The microbial DNA was used to establish metagenomics shotgun sequencing libraries with 400 bp inserts at Personal Biotechnology Co., Ltd., located in Shanghai, China, using the Illumina TruSeq Nano DNA LT Library Preparation Kit (Kuang et al., 2023). The Illumina NovaSeq platform (Illumina, United States) was employed for sequencing the libraries using a PE150 strategy.

### 2.4 Metagenomic analysis

Clean genomic assembly data were acquired from raw data using FASTP software (v18.0) (Chen S. et al., 2018). Megahit (v1.1.2) and MetaGeneMark (v3.38) were used for genetic assembly and prediction (Zhu et al., 2010; Li et al., 2015). To cluster the CDS sequences of all samples, mmseqs2 was employed in “easy-cluster” mode, with a protein sequence identity threshold of 0.90 and a coverage of 90% for the shorter contig (Steinegger and Söding, 2017). To analyze the gene abundance, high-quality reads from each sample were aligned to predicted gene sequences using Salmon in ‘quasi-mapping-based’ mode (Patro et al., 2015). Abundance values in the metagenomes were normalized using copies per kilobase per million mapped reads (CPM). Non-redundant gene function was determined by annotating with mmseqs2 in the search mode against the KEGG, EggNOG and CAZy protein databases, respectively. EggNOG and GO were obtained using EggNOG-mapper (v2) (Cantalapiedra et al., 2021). The GO ontology was procured via map2slim from www.metacpan.org, and KO was acquired through KOBAS (Bu et al., 2021).

### 2.5 Antimicrobial susceptibility tests

*Enterococcus casseliflavus* was identified as the dominant bacterium in diapause larvae of *C. bilineata tsingtauica* through metagenomic analysis. To explore the nutritional function of *E. casseliflavus* affecting on larvae, the antimicrobial susceptibility tests were carried out to screen the most effective antibiotic against *E. casseliflavus*. The following antibiotics were used in the experiment: ampicillin (AMP), vancomycin (VAN), ciprofloxacin (CIP), gentamicin (GM), erythromycin (EM), levofloxacin (LVX), chloromycetin (CHL) and tetracycline (TC) (Liang et al., 2022; Zhang and Ju, 2023). The strain of *E. casseliflavus* was obtained from China General Microbiological Culture Collection Center (CGMCC)^1^ and cultured according to the instructions. The solid media were prepared with 30 g trypticase soy broth, 15 g agar, and 1 L double distilled water, sterilized at 121°C for 15 min. Evenly applied the solution of *E. casseliflavus* onto the solid media, cultured at 37°C for 12 h. The colony count reached a period of logarithmic growth. Place the susceptibility paper bought from Shanghai Ebaiju Economic and Trade Co. LTD (Shanghai, China) at the center of the media. Incubate the media at 37°C for 24 h. Measure the bacteriostatic diameter in each medium using a vernier caliper, and carefully determine the clarity of the bacteriostatic zones. Each antibiotic treatment was repeated for six times.

### 2.6 Statistical analysis

Linear discriminant analysis effect size (LEfSe) was performed to detect differentially abundant taxa and functions across groups using the default parameters based on the taxonomic and functional profiles of non-redundant genes (Segata et al., 2011). Bray-Curtis distance metrics were utilized to perform beta diversity analysis, examining compositional and functional differences in microbial communities among samples. The results were presented through principal coordinate analysis (PCoA), non-metric multidimensional scaling (NMDS) and unweighted pair-group method with arithmetic means (UPGMA) hierarchical clustering (Bray and Curtis, 1957; Ramette, 2007). Antimicrobial susceptibility test results were reported as mean and standard errors of mean (Mean ± SEM). The diameters of inhibition zones treated with eight antibiotics were analyzed through the SPSS 25.0 software (Chicago, IL, United States), employing a one-way analysis of variance (ANOVA) test. Tukey’s test was used to determine statistically significant differences among the treatments at *p* < 0.05. All data were first analyzed by using Levene and Kolmogorov–Smirnov tests to determine the homogeneity of variance and normality, respectively. Transformed data was used for analysis if the raw data did not confirm the homogeneity.

## 3 Results

### 3.1 Quality analysis of metagenomic sequencing data from diapause larvae

Nine metagenomic DNA libraries were sequenced on the Illumina NovaSeq platform, constructed from the control group (D0) and the diapause groups (D14 and D28). As shown in Table 1, the number of total reads ranged from 41,811,872 to 50,374,154, with a base count ranging from 6,271,780,800 to 7,556,123,100. The GC (%) of the control and diapause groups ranged from 43.86 to 47.3% and from 42.94 to 49.59%, respectively (see Table 1). The Ns (%) presented in Table 1 indicate that fuzzy bases were found in both groups. Moreover, it is noteworthy that all Q20s (%) and Q30s (%) from both control and diapause groups exceeded 95 and 90%, respectively (refer to Table 1). The sequencing data met the prescribed criteria, as evidenced by statistical distributions for sequencing quality (Supplementary Figure S1A), mean mass (Supplementary Figure S1B), base species content (Supplementary Figure S1C), and GC content (Supplementary Figure S1D). Consequently, it is viable for further analysis.

**Table 1:** Sequencing data of intestinal microbial metagenomics in the dipause larvae of soybean hawkmoth *Clanis bilineata tsingtauica.*

Sample	Total number of reads	Total number of bases (bp)	*N* (%)	GC (%)	Q20 (%)	Q30 (%)
D0-1	44,864,170	6,729,625,500	0.00254	42.25	97.53	92.97
D0-2	45,321,928	6,798,289,200	0.00081	42.69	97.13	92.23
D0-3	50,374,154	7,556,123,100	0.00316	46.20	97.92	93.83
D14-1	41,811,872	6,271,780,800	0.00321	51.20	98.18	94.41
D14-2	41,988,150	6,298,222,500	0.00317	54.49	98.18	94.40
D14-3	45,431,020	6,814,653,000	0.00097	53.49	97.35	92.88
D28-1	43,497,680	6,524,652,000	0.00088	48.26	97.24	92.61
D28-2	47,194,260	7,079,139,000	0.00074	48.96	96.63	91.37
D28-3	42,410,840	6,361,626,000	0.00104	50.41	97.61	93.47

D0 - Control (diapause day 0); D14 - Diapause day 14; D28 - Diapause day 28; N (%) represents the percentage of fuzzy bases in the total number of bases; GC (%) represents the percentage of G and C bases in the total number of bases; Q20 (%) and Q30 (%) indicate the percentage of the bases with a determination accuracy above 99 and 99.9%, respectively, in relation to the total number of bases.

### 3.2 Diapause time reduces the abundance of gut dominant bacteria *Enterococcus*

**Figure 1:**
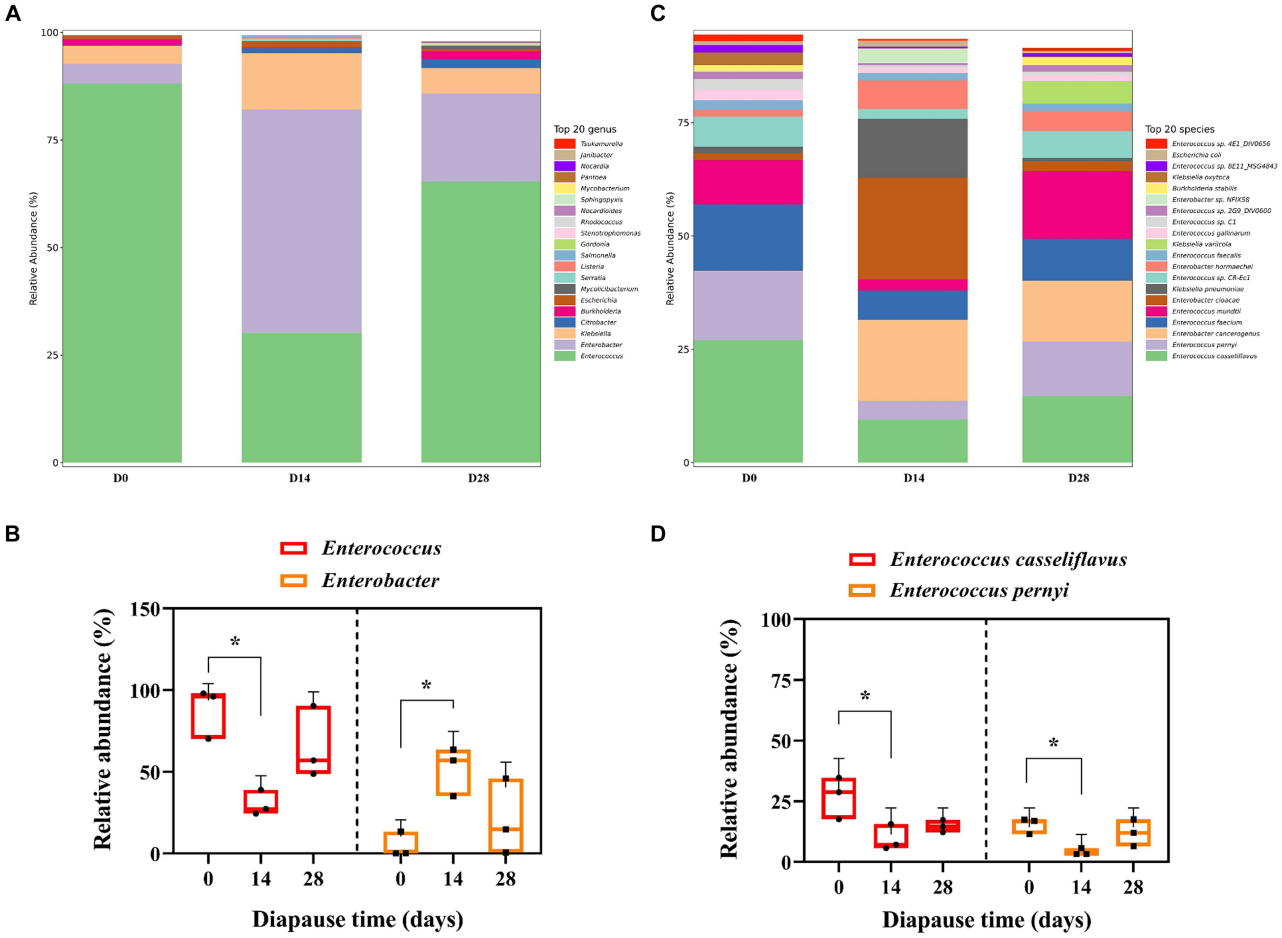
Effects of diapause time on the compositions of gut microbiota in the dipause larvae of soybean hawkmoth *Clanis bilineata tsingtauica*. The compositions **(A)** and the relative abundance (RA) **(B)** of *Enterococcus* and *Enterobacter* at the genus level. The compositions **(C)** and the RA **(D)** of *Enterococcus casseliflavus* and *Enterococcus pernyi* at the species level. Each histogram distinguishes taxa by color, and the ordinate represents the RA of each taxon. The longer the column, the higher the RA of the taxa. Different lowercase letters denote significant difference among diapause time by Tukey test at *p* < 0.05.

The predominant bacterial genera found in the midgut of diapause larvae of soybean hawkmoth were *Enterococcus* and *Enterobacter*, with the primary species being *Enterococcus casseliflavus* and *Enterococcus pernyi* (Figure 1 and Supplementary Figure S2). The relative abundance (RA) of *Enterococcus* significantly decreased by 65.74% on D14 compared to D0 controls (*p* < 0.05, *F* = 9.71, df = 2; Figure 1B). However, the RA of *Enterobacter* on D14 was significantly higher than the controls by 1031.10% (*p* < 0.05, *F* = 6.38, df = 2; Figure 1B). A similar trend was noticed in the RA of *E. casseliflavus* and *E. pernyi* over diapause time (Figure 1D). Specifically, both of *E. casseliflavus* and *E. pernyi* displayed a significantly lower RA on D14 in comparison to the control group (*p* < 0.05, *F* = 6.60, df = 2; *p* < 0.05, *F* = 6.81, df = 2; Figure 1D).

**Figure 2:**
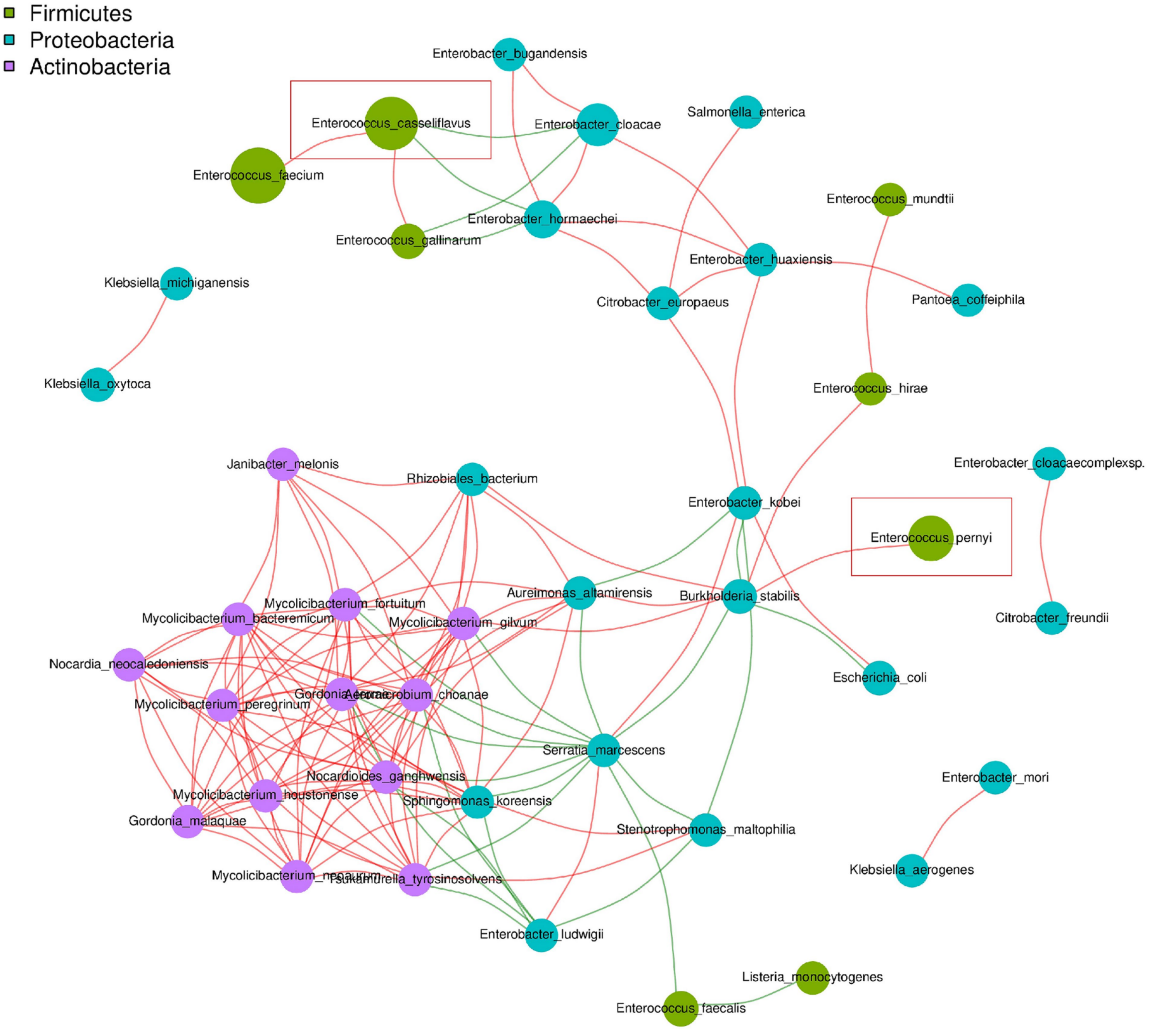
Map of the dominant microbial association network in the dipause larvae of soybean hawkmoth *C. bilineata tsingtauica* at species level. The dominant genera represented by nodes are marked with different colors. The connection between the nodes indicates a correlation between two species. Red lines indicate a positive correlation and green lines indicate a negative correlation.

As shown in Figure 2, *E. casseliflavus* exhibited a positive association with *Enterococcus faecium* and *Enterococcus gallinarum*, and a negative association with *Enterobacter cloacae* and *Enterobacter hormaechei*. By contrast, *E. pernyi* displayed a positive correlation only with *Burkholderia stabilis*.

### 3.3 Diapause time increases the diversity of larval gut microbiome

**Figure 3:**
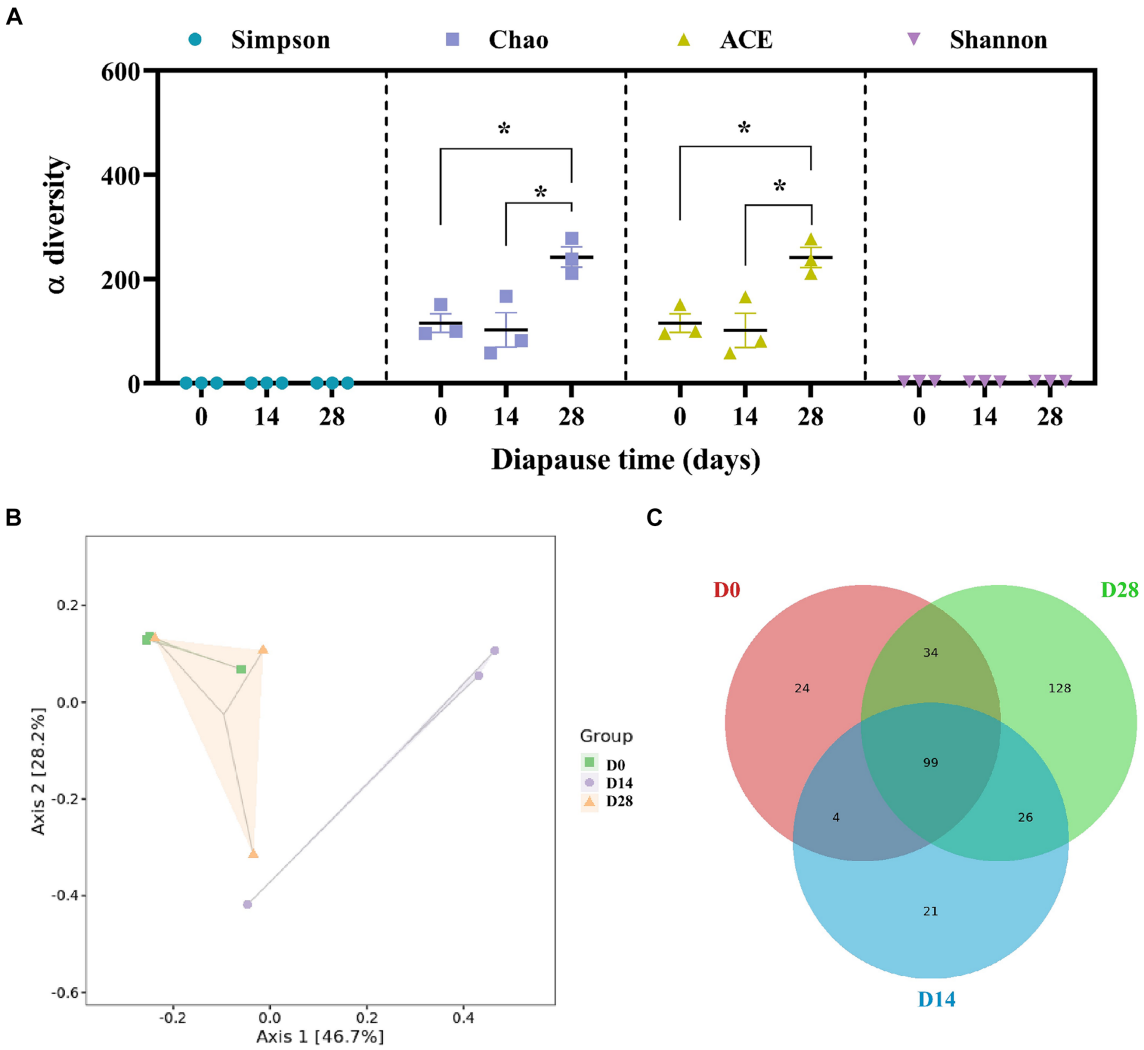
Effects of diapause time on the diversity and species difference of gut microbiota in the diapause larvae of *C. bilineata tsingtauica*. α-diversity **(A)**, PCoA analysis **(B)** and Venn figure **(C)** of gut microbiota. Different lowercase letters denote significant differences among diapause time by Tukey test at *p* < 0.05.

There was a significant increase in Chao and ACE (abundance-based coverage estimator) on D28 compared to D0 (*p* < 0.05, *F* = 10.03, df = 2; *p* < 0.05, *F* = 10.13, df = 2; Figure 3A). This suggests that the species richness of gut microbiota increased in diapause larvae on D28. PCoA demonstrated a clear separation of gut microbiota profiles between D14 and D0, D28 (Figure 3B). The cluster heatmap of the top 50 differential species, the LEfSe analysis and the UPGMA hierarchical clustering analysis all provided supportive evidence for PCoA (Supplementary Figure S3). A total of 99 common microbial species among groups were revealed in the Venn diagram presented in Figure 3C.

### 3.4 Diapause time up-regulates larval amino acid metabolic pathways

**Figure 4:**
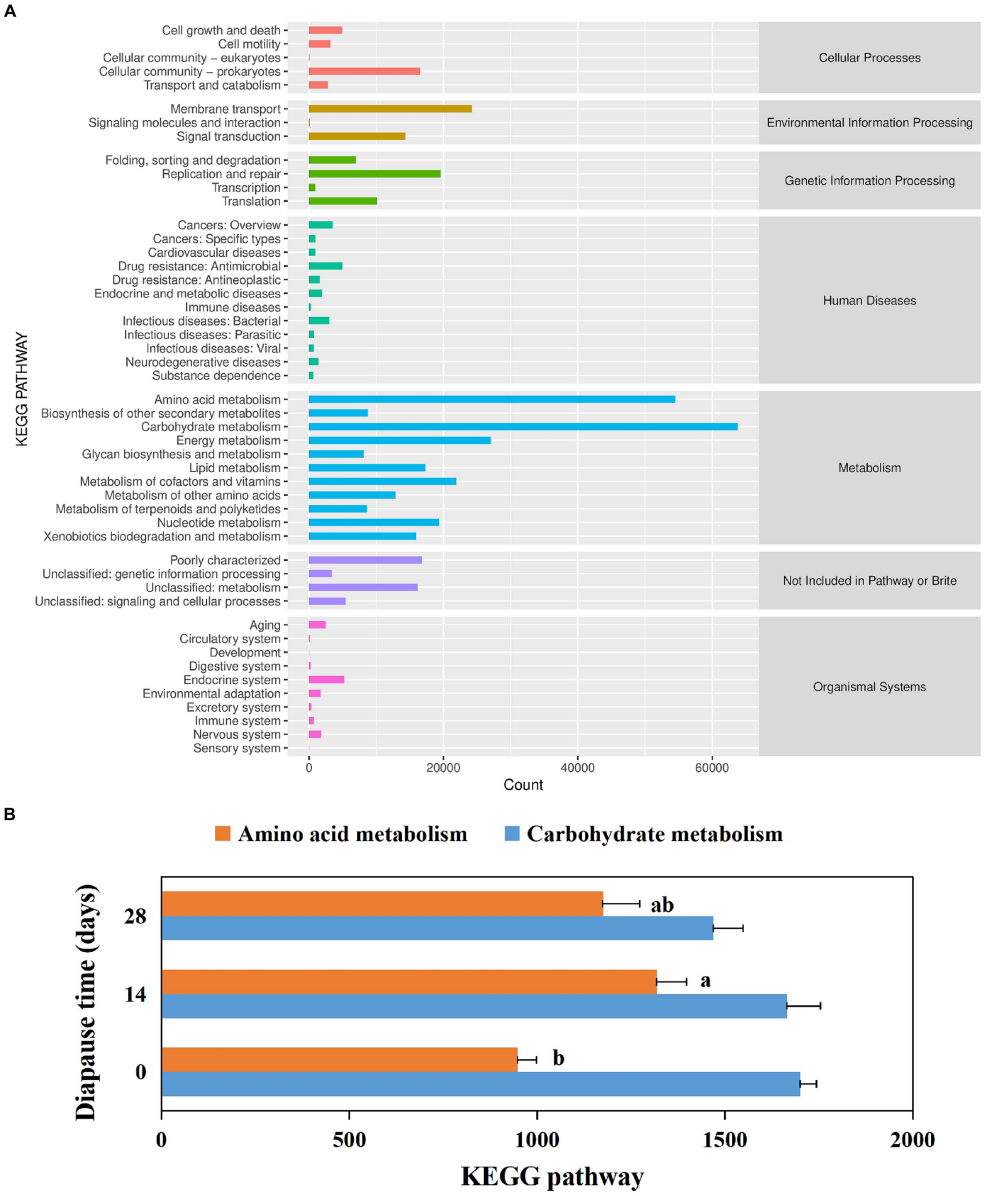
Analysis of annotated KEGG metabolic pathways in the diapause larvae of *C. bilineata tsingtauica* **(A)**. Effects of diapause time on the KEGG metabolic pathways in the dipause larvae **(B)**. The horizontal coordinates represent the number of proteins annotated to the corresponding pathways, and the vertical coordinates correspond to pathways at KEGG level 2. The classification of pathways at KEGG level 1 is shown on the right. Different lowercase letters denote significant differences among diapause time by Tukey test at *p* < 0.05.

Larval gut microbiota function was mainly focused on metabolism (see Figure 4A). The top five pathways included carbohydrate metabolism, amino acid metabolism, energy metabolism, metabolism of cofactors and vitamins, and nucleotide metabolism (Figure 4A). In addition, the annotated amino acid metabolic pathways were significantly higher in diapause larvae on D14 than in controls (*p* < 0.05, *F* = 11.98, df = 2; Figure 4B).

**Figure 5:**
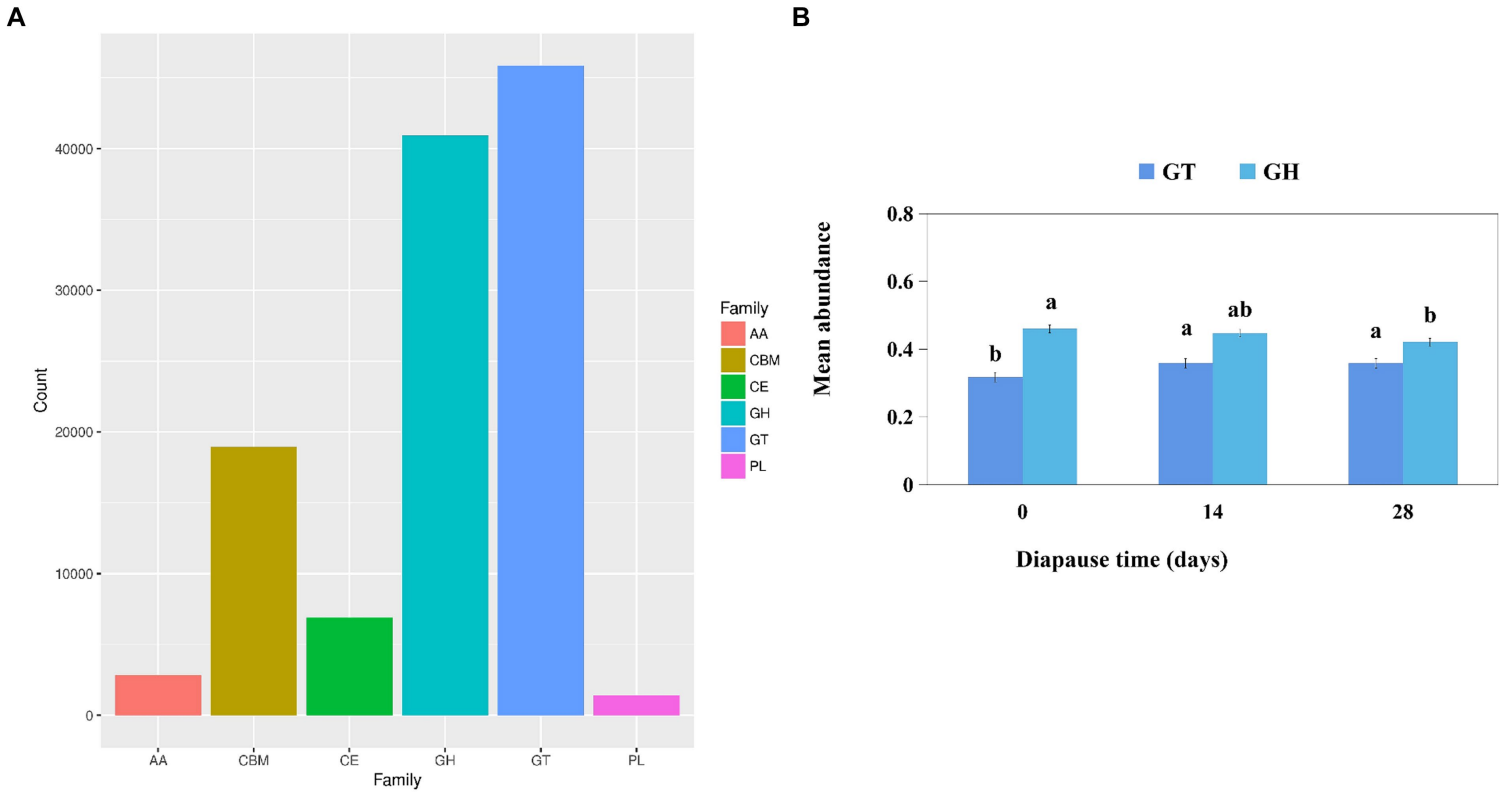
Analysis of the function module annotation of carbohydrate-active enzymes (CAZys) **(A)**. Effects of diapause time on the mean abundance of CAZys **(B)**. Different lowercase letters denote significant differences among diapause time by Tukey test at *p* < 0.05.

Functional module annotation of carbohydrate-active enzymes (CAZy) primarily focused on glycosyl transferases (GT) and glycoside hydrolases (GH) (Figure 5A). The mean abundance of larval GT was significantly higher on D14 and D28 compared to the controls (*p* < 0.05, *F* = 7.89, df = 2; *p* < 0.05, *F* = 7.07, df = 2; Figure 5B). Additionally, the level of larval GH was significantly lower on D28 compared to the controls (*p* < 0.05, *F* = 8.53, df = 2; Figure 5B).

### 3.5 Tetracycline is the most effective antibiotic against *E. casseliflavus*

**Figure 6:**
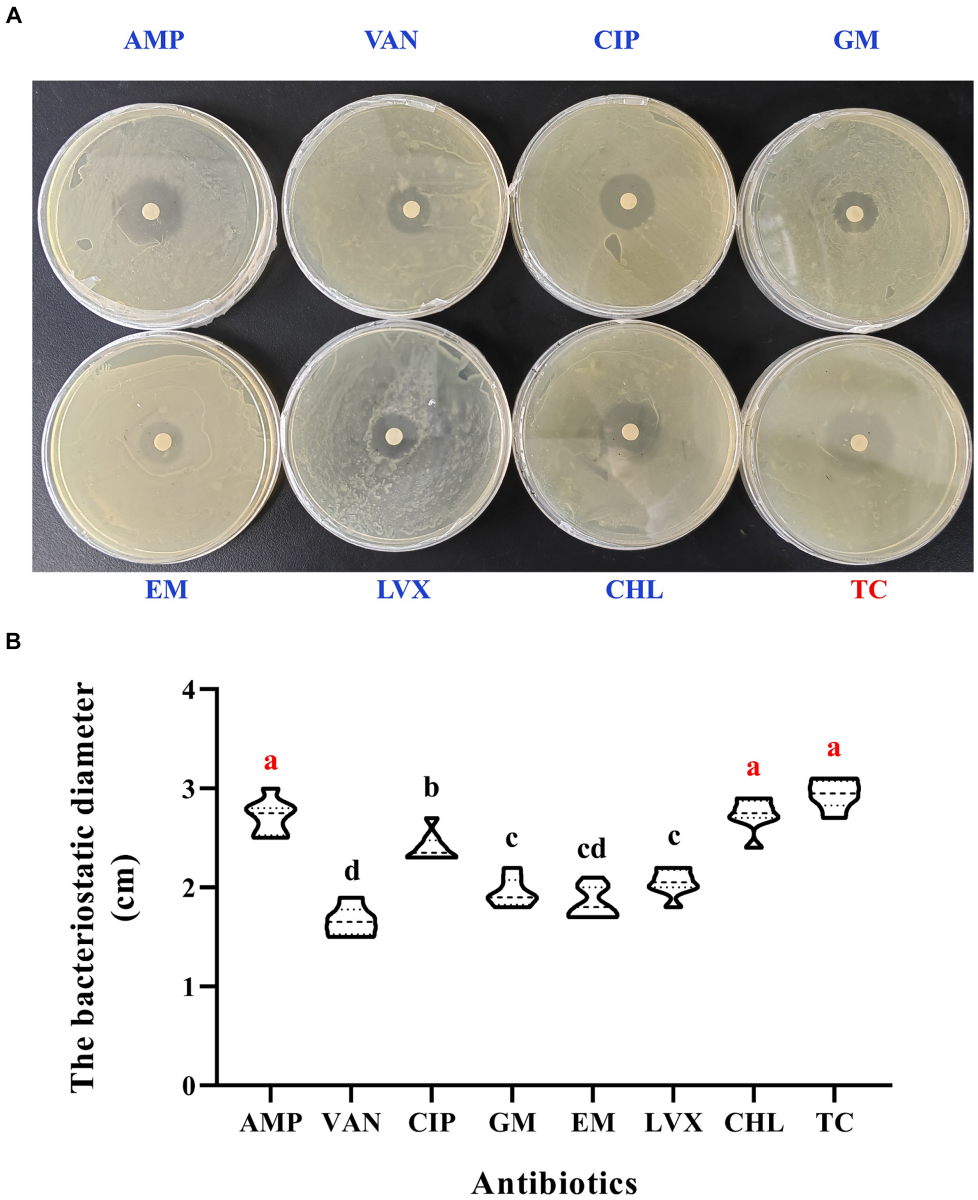
Screening of antibiotics targeted on *E. casseliflavus*. The susceptibility paper tests **(A)**. The bacteriostatic diameter in the media treated with different antibiotics **(B)**. AMP, ampicillin; VAN, vancomycin; CIP, ciprofloxacin; GM, gentamicin; EM, erythromycin; LVX, levofloxacin; CHL, chloromycetin; TC, tetracycline. Different lowercase letters denote significant differences among different antibiotics by Tukey test at *p* < 0.05.

Eight antibiotics were evaluated for their effectiveness against *E. casseliflavus*, the dominant bacterium in the midgut of diapause soybean hawkmoths. TC, CHL and AMP have been determined as the most efficient antibiotics against *E. casseliflavus* in diapause larvae (see Figure 6). The mediums treated with these three antibiotics demonstrated more distinct bacteriostatic zones in comparison to others (*p* < 0.05, *F* = 60.75, df = 7; Figure 6A). The bacteriostatic diameter resulting from treatments with TC, CHL and AMP significantly exceeded that of the other five antibiotics (*p* < 0.05, F = 60.75, df = 7; Figure 6B).

## 4 Discussion

As reported by Jing et al. (2020), the gut microbiota played a vital role in allocating and mobilizing nutrients in insects during diapause. This study employed metagenomic analysis to identify the dominant gut bacteria in the diapause larvae of soybean hawkmoths from Jiangsu Province. The dominant genera of gut bacteria found in diapause larvae were determined to be *Enterococcus* and *Enterobacter*. Our previous study on 16S rRNA sequencing analysis showed that *Enterococcus* and *Stenotrophomonas* were the predominant taxa at the genus level (Qian et al., 2023b). Thus, *Enterococcus* was certainly evidenced to be the predominant genus in the diapause larvae of soybean hawkmoths, while *Enterobacter* belongs to *Proteobacteria*, it was generally involved in the immunity and pathogenicity of insects. Xie et al. (2023) also reported that *Enterococcus* was the predominant genus found in soybean hawkmoths from Henan Province. Similar findings have been observed in other Lepidopteran species. For example, Kong et al. (2023) found that *Enterococcus* spp. were the predominant gut bacteria in *Galleria mellonella* larvae, with *Enterobacter* spp. being dominant in the pupae. However, Lv and Liu (2009) reported that *Staphylococcus* and *Bacillus* were the dominant bacterial genera in diapause soybean hawkmoths from Shandong Province by combining a combination of morphological identification and 16S DNA sequencing. Differences in results may be stem from variations in geographic populations and genetic differentiation, as well as advancements in next-generation sequencing (NGS) techniques. In recent years, metagenomic analysis has been widely used to study the gut microbiome of numerous species. The metagenomic analysis conducted in this study indicated that *Enterococcus casseliflavus* and *Enterococcus pernyi*, both belonging to the *Enterococcus* genus, were the dominant gut bacterial species detected in diapause larvae of soybean hawkmoth. It represents the first identification of such gut bacteria in soybean hawkmoths. This study advances our understanding of the interaction between gut microbes and insect hosts by identifying the dominant bacterium from genus to species levels.

The diapause stage is critical for insect development and presents challenges for both hosts and microbial partners (Dittmer and Brucker, 2021). This study examined how diapause time impacts composition, diversity and functionality of gut microbiomes present in soybean hawkmoths. The relative abundance of *Enterococcus*, including *E. casseliflavus* and *E. pernyi*, initially decreased on diapause D14 and slightly increased on D28. For the species diversity analysis, D14 was significantly separated from both D0 and D28, but D0 and D28 appear to overlap considerably. It suggested that the larvae may potentially survive harsh diapause conditions with the assistance of their dominant gut bacteria. *Enterococcus* is a predomiant gut commensal bacterium that plays a crucial role in the physiology and health of the major economic agricultural insect, *B. mori* (Zhang et al., 2022). *Enterococcus faecium* has been demonstrated to regulate the developmental genes in honey bees (Du et al., 2021). Kong et al. (2023) provided evidence that *Enterococcus* spp. significantly contributes to insect metamorphosis. Furthermore, Chao and ACE showed an increase with diapause time, indicating a greater abundance of gut microbiota in late diapause larvae.

Gut microbiota reportedly contributes significantly to insects’ nutritional metabolism, digestive function and endocrine signals (Fraune and Bosch, 2010; Dittmer and Brucker, 2021). Gut microbes are capable of consistently supplying insect hosts with some nutrients (e.g., EAAs, carbohydrates) which can boost metabolic production (Fraune and Bosch, 2010). During diapause, the axenic larvae of parasitic wasp *Nasonia vitripennis* consistently maintained lower levels of glucose and glycerol than conventional larvae (Dittmer and Brucker, 2021). In this study, the function of larval gut microbiota was primarily focused on metabolism, particularly in carbohydrate metabolism and amino acid metabolism. Chen B. et al. (2018) used shotgun metagenomic sequencing techniques to reveal that the gut microbiome of *B. mori* equally emphasizes the metabolism of both carbohydrates and amino acids. Additionally, the metabolic pathways of amino acids in the gut microbiota of soybean hawkmoths were up-regulated with extended diapause time, as indicated by the annotated data. An opposite trend was observed between the abundance of the dominant gut bacterium *Enterococcus* and the amino acid metabolic pathways with diapause time. Similarly, the relative abundance of *Enterococcus* in *B. mori* had a significant correlation with the concentration of amino acids (Li et al., 2022). Liang et al. (2022) reported that *E. casseliflavus* is capable of producing L-tryptophan in *B. mori*. It suggested that *Enterococcus* may upregulate amino acid metabolic pathways to maintain the nutritional balance in diapause soybean hawkmoths.

Based on the previous non-target metabolomics analysis on diapause soybean hawkmoths, it showed that the most enriched pathways were mainly those involved in amino acid metabolism. The differential metabolite D-glutamine was found to be involved in arginine biosynthesis pathways (Qian et al., 2023b). D-glutamine has been reported to assist in nutrient delivery (particularly amino acids) for the metabolism during insects’ immune response to adverse conditions, such as low temperatures or food restrictions (Dolezal et al., 2019). Moreover, the content of arginine in diapause larvae of soybean hawkmoths (Qian et al., 2023b) also exhibited an opposite alteration to the abundance of dominant gut bacterium *E. casseliflavus*. Therefore, it is speculated that soybean hawkmoths probably up-regulate arginine metabolism by reducing the abundance of *E. casseliflavus*, thus ensuring their nutritional balance during diapause.

*E. casseliflavus*, the dominant gut bacterium in diapause larvae of soybean hawkmoths, exhibits antibiotic resistance, complicating the investigation of its interaction with insect hosts. Tetracycline (TC), chloromycetin (CHL) and ampicillin (AMP) were screened as the most effective antibiotics for targeting it in this study. These results align with those found in other Lepidopteran insects. For example, Li (2022) applied TC and AMP to treat *E. casseliflavus* in *Cnaphalocrocis medinalis*, and TC was used to treat *Tetranychus truncatus* (Yang et al., 2020). Next, the mixed liquor of TC, CHL and AMP will be used to determine the nutritional function of *E. casseliflavus* affecting on *C. bilineata tsingtauica*. It will be helpful to the farming industry of *C. bilineata tsingtauica* via the manual intervention of intestinal microecology, and provide scientific basis for the resource exploitation and utilization of edible insects.

## 5 Conclusion

In summary, *Enterococcus casseliflavus* was identified as the dominant bacterium in diapause larvae of *C. bilineata tsingtauica*, with the gene function focusing on carbohydrate metabolism and amino acid metabolism. In addition, we demonstrated that diapause time reduced the relative abundance of *E. casseliflavus*, while concurrently enhancing the relative abundance of amino acid metabolic pathways. Building on our previous non-target metabolomics research, it is possible that diapause larvae of *C. bilineata tsingtauica* may up-regulate their arginine metabolism by reducing the levels of *E. casseliflavus* to maintain their nutritional balance. This not only extends our comprehension of dominant gut bacteria from the genus to the species level, but also offers an initial investigation of gene functionality in soybean hawkmoths. Furthermore, TC was screened as the most effective antibiotic target for *E. casseliflavus* in soybean hawkmoths. Nevertheless, further verification is required for the effect of TC *in vivo* experiments and the appropriate treatment concentrations. Next, we will systematically measure the gut microbiota of soybean hawkmoths from newly hatched larvae to adults, and investigate the interaction mechanism between *E. casseliflavus* and insect hosts.

## Data availability statement

The original contributions presented in the study are publicly available. This data can be found here: https://ncbi.nlm.nih.gov/bioproject/PRJNA1043846.

## Author contributions

LQ: Conceptualization, Funding acquisition, Investigation, Writing – original draft. YW: Investigation, Methodology, Writing – original draft. PD: Investigation, Methodology, Writing – original draft. JZ: Investigation, Writing – original draft. YQ: Data curation, Investigation, Writing – original draft. ZL: Data curation, Investigation, Writing – original draft. HL: Project administration, Writing – review & editing. FC: Supervision, Writing – review & editing.

## Footnotes

^1^https://cgmcc.net
