# Supplementary material for: Enterococcus casseliflavus regulates amino acid metabolism in edible insect Clanis bilineata tsingtauica: a functional metagenomics study
Source: Front Microbiol. 2024 Mar 25;15:1343265. doi: 10.3389/fmicb.2024.1343265 (PMC10999662; doi:10.3389/fmicb.2024.1343265)
Supplement: Supplementary file 1 [file Data_Sheet_1.doc]

***Supplementary Material***

**Supplementary Figure S1** Quality analysis of of the intestinal microbial metagenomic sequencing data in soybean hawkmoths larvae, including the statistical distribution of sequencing quality (A), mean mass distribution (B), content distribution of base species (C), and GC content distribution (D).


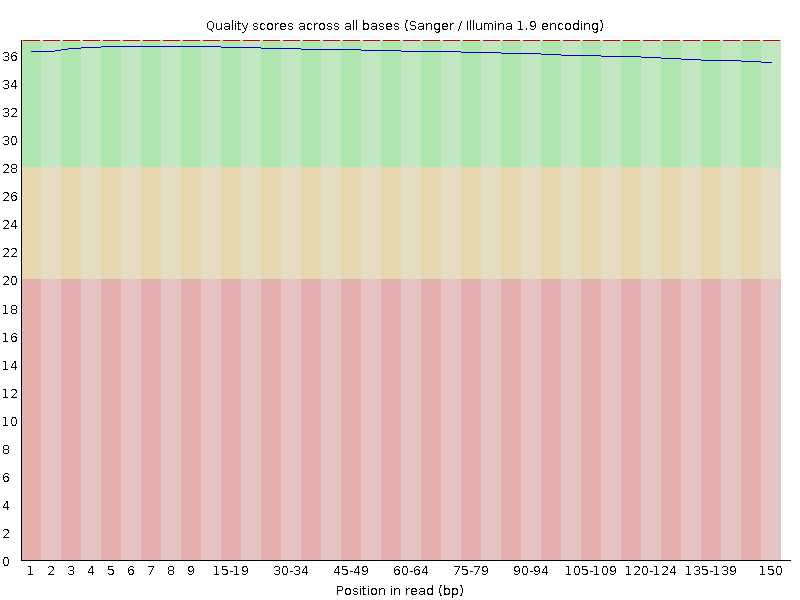

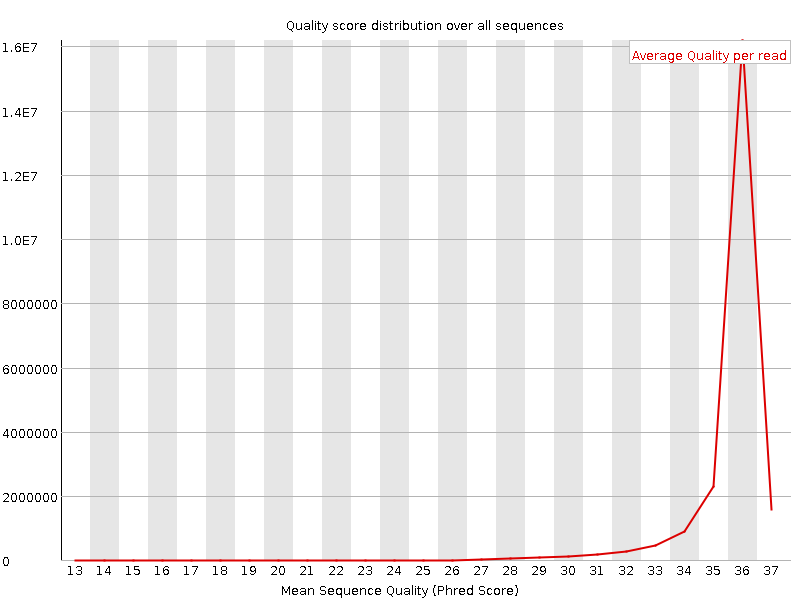

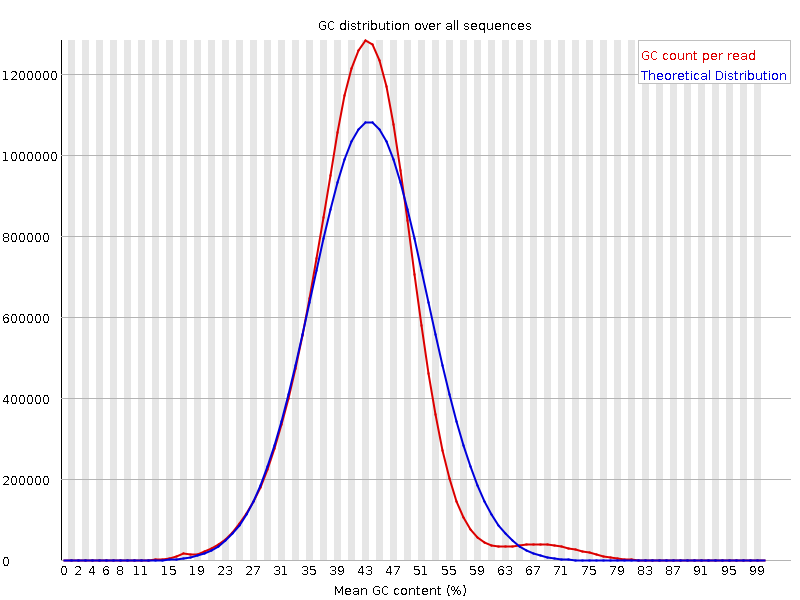

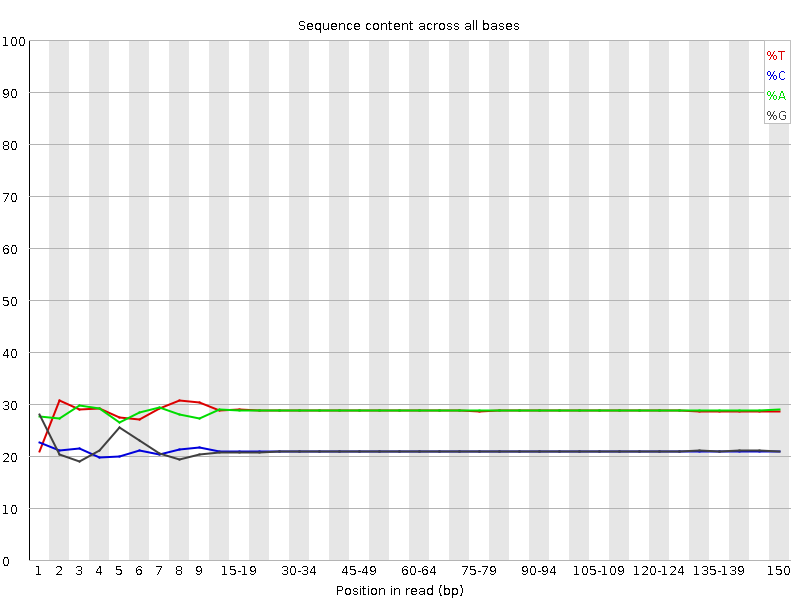


**(A)**

**(B)**

**(C)**

**(D)**


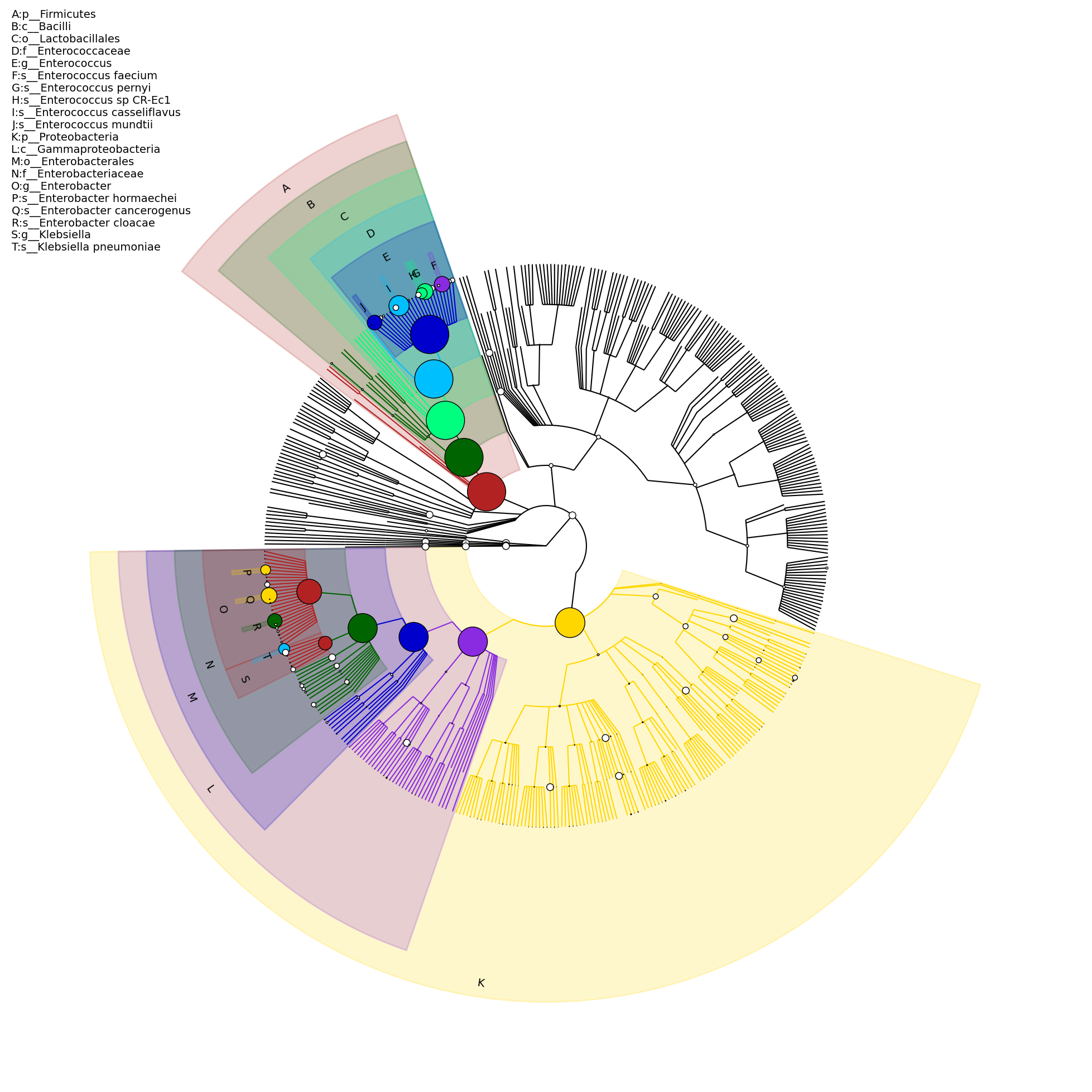


**Supplementary Figure S2** The tree graph of sample classification level based on GraPhlAn. From the inner to the outer circles, the hierarchical relationship of all taxonomic units (represented by nodes) from phylum to species in the sample population is shown in sequence. The size of the nodes corresponds to the average relative abundance of the taxonomic unit. The top 20 taxonomic units in relative abundance are also identified by letters in the figure. The shadow on the letter is the same color as the corresponding node.

**
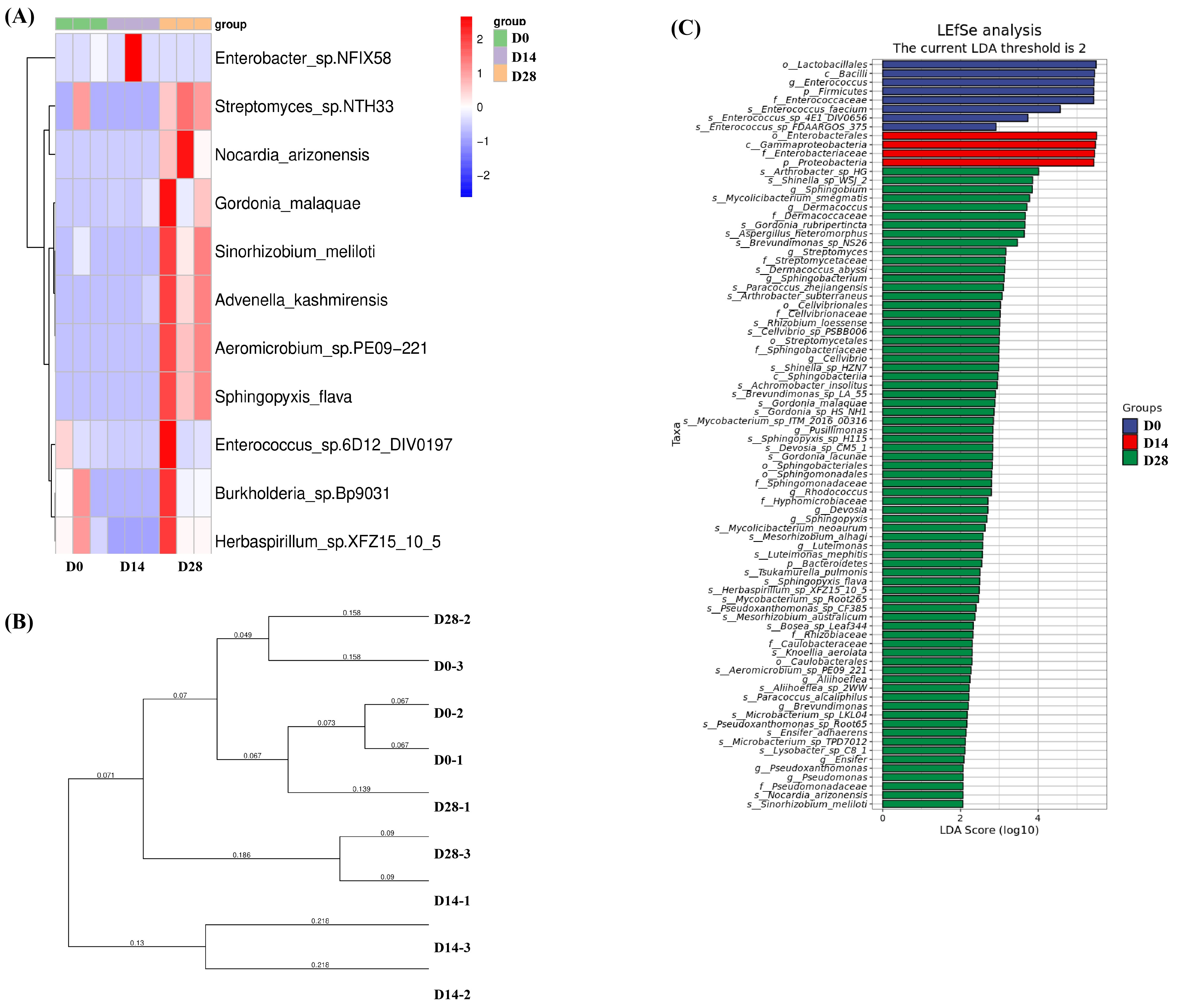
**

**Supplementary Figure S3** Effect of diapause time on the species difference of soybean hawkmoths larvae. (A) The cluster heatmap of TOP 50 differential species. Red represents species with higher abundance and blue represents species with lower abundance. (B) Unweighted pair-group method with arithmetic means (UPGMA) hierarchical clustering analysis diagram. (C) Linear discriminant analysis Effect Size (LEfSe) analysis diagram. Taxonomic cladistics shows the taxonomic hierarchy of the major taxa, from phylum to species.
